# Improving Real-Time Brain State Classification of Motor Imagery Tasks During Neurofeedback Training

**DOI:** 10.3389/fnins.2020.00623

**Published:** 2020-06-24

**Authors:** Epifanio Bagarinao, Akihiro Yoshida, Kazunori Terabe, Shohei Kato, Toshiharu Nakai

**Affiliations:** ^1^Brain & Mind Research Center, Nagoya University, Nagoya, Japan; ^2^Neuroimaging and Informatics Group, National Center for Geriatrics and Gerontology, Obu, Japan; ^3^Graduate School of Engineering, Nagoya Institute of Technology, Nagoya, Japan; ^4^Department of Radiology, Osaka University Graduate School of Dentistry, Osaka, Japan

**Keywords:** real-time fMRI, motor imagery, neurofeedback, support vector machines, incremental training, brain state, learning

## Abstract

In this study, we investigated the effect of the dynamic changes in brain activation during neurofeedback training in the classification of the different brain states associated with the target tasks. We hypothesized that ongoing activation patterns could change during neurofeedback session due to learning effects and, in the process, could affect the performance of brain state classifiers trained using data obtained prior to the session. Using a motor imagery paradigm, we then examined the application of an incremental training approach where classifiers were continuously updated in order to account for these activation changes. Our results confirmed our hypothesis that neurofeedback training could be associated with dynamic changes in brain activation characterized by an initially more widespread brain activation followed by a more focused and localized activation pattern. By continuously updating the trained classifiers after each feedback run, significant improvement in accurately classifying the different brain states associated with the target motor imagery tasks was achieved. These findings suggest the importance of taking into account brain activation changes during neurofeedback in order to provide more reliable and accurate feedback information to the participants, which is critical for an effective neurofeedback application.

## Introduction

Real-time functional magnetic resonance imaging (fMRI), coupled with machine learning algorithms, has enabled the real-time identification of different brain states during fMRI scans (LaConte et al., 2007; LaConte, 2011; Shibata et al., 2011; Sitaram et al., 2011; Bagarinao et al., 2018). Using support vector machines (SVM), a type of supervised machine learning algorithm, LaConte et al. (2007) demonstrated the first real-time decoding of brain states corresponding to the left and right button presses task. In the same study, they also demonstrated the classifier’s ability to decode other cognitive and emotional states, albeit in a small number of participants. This approach was later extended by Sitaram et al. (2011) to the online classification and feedback of multiple emotional states. Shibata et al. (2011) used sparse logistic regression to decode target activation patterns from localized brain regions and used neurofeedback to induce perceptual learning.

Recently, we employed a multivariate pattern analysis using SVM to demonstrate the importance of feedback information in improving volitional recall of brain activation patterns during motor imagery training (Bagarinao et al., 2018). Specifically, we examined the effect of neurofeedback in recalling activation patterns associated with motor imagery tasks. For this purpose, participants underwent extended motor imagery task training consisting of two scanning sessions, one with feedback and the other without feedback. Consistency in recalling motor imagery relevant activation patterns was assessed using SVM. The results clearly showed that with feedback information, participants were able to recall relevant activation patterns significantly better than without feedback. For the training with feedback, we used data obtained from an initial scan to train SVMs, which were later used in the succeeding feedback runs (LaConte, 2011). We observed that the SVMs’ classification performance tended to decrease with each feedback run.

One of the implicit assumptions in brain state classification studies using supervised learning algorithms is the stationarity of the measured system (i.e., the brain) (Vapnik, 1999; Hastie et al., 2009). However, for most neurofeedback studies, the goal of training is for participants to learn the target tasks. This can be in the form of either up- or down-regulating activity in circumscribed brain regions (DeCharms et al., 2004; deCharms et al., 2005; Caria et al., 2007; Zotev et al., 2011, 2018; Hamilton et al., 2016; Sherwood et al., 2016), inducing specific patterns of brain activity (Shibata et al., 2011; Amano et al., 2016; Cortese et al., 2016, 2017; Koizumi et al., 2016), or enhancing connectivity between regions (Koush et al., 2013, 2017; Kim et al., 2015). As demonstrated by earlier studies, neurofeedback training can change functional brain networks (Haller et al., 2013) and can alter the profile of connectivity patterns of specific brain regions, for example, in the right inferior gyrus (Rota et al., 2011) as well as in the insular cortex (Lee et al., 2011). All of these entail dynamic changes in the brain’s activity if the participant has to learn the target tasks.

For neurofeedback to be effective, the feedback information also needs to be reliable and representative of the ongoing brain activity or activation pattern that was meant to be volitionally controlled. Otherwise, the feedback signal would not be of help to participants in actually self-regulating their brain activity. This can be clearly seen in neurofeedback studies that used sham control (deCharms et al., 2005; Rota et al., 2009; Caria et al., 2010; Zotev et al., 2018). Under the sham condition, the feedback signal provided to the participants does not represent actual activation. Findings using sham control have clearly demonstrated that the desired effect is not usually achieved, suggesting the importance of having the right feedback information for neurofeedback training to be effective. To account for the changing activation pattern that could be driven by learning effects and to be able to provide relevant feedback information, the system itself must be able to adapt as the training progresses.

In this study, we hypothesized that during neurofeedback training, dynamic changes in activation patterns could occur as participants learned to perform the target tasks. To account for these changing activation patterns, we examined whether continuously updating trained classifiers after every feedback scan could improve the classifiers’ performance and thus provide a more accurate feedback information to the participants. For this, we used a brain–machine interface (BMI) system that employed a motor imagery paradigm coupled with real-time fMRI based neurofeedback (Bagarinao et al., 2018). The choice of motor imagery, a covert cognitive process where an action is mentally simulated but not actually performed (Grèzes and Decety, 2000; Hétu et al., 2013), is motivated by its potential as an effective neurorehabilitation tool to improve motor functions (Jackson et al., 2001; Zimmermann-Schlatter et al., 2008; Malouin and Richards, 2013). We used linear SVMs to classify brain states associated with different motor imagery tasks and evaluated its performance during feedback runs.

## Materials and Methods

### Participants

We recruited 30 healthy young participants (15 males and 15 females), ranging in age from 20 to 25 years old (mean age = 21.7 years, standard deviation = 1.3 years) for this study. All participants had no history of neurological or psychiatric disorders, right handed as indexed by a handedness inventory test for Japanese (Hatta and Hotta, 2008), and with Mini-Mental State Examination score greater than 26. This study was approved by the Institutional Review Board of the National Center for Geriatrics and Gerontology of Japan. All participants gave written informed consent before joining the study.

### Experimental Paradigm and Tasks

**FIGURE 1:**
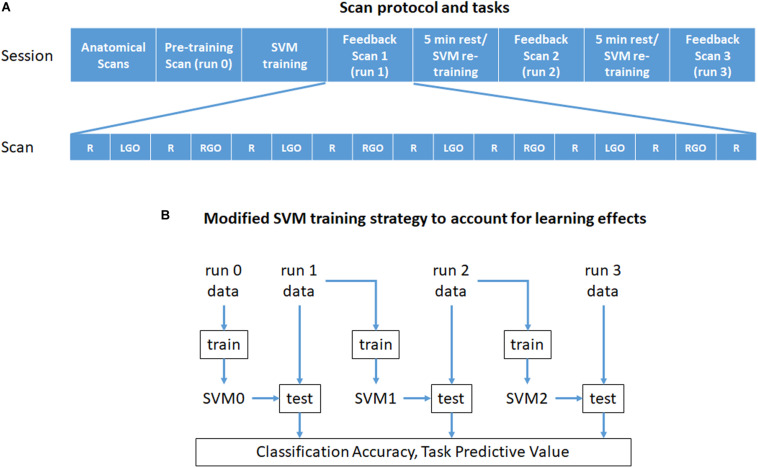
Outline of **(A)** the experiment protocol and tasks and **(B)** the modified training strategy used in this study. Each scan block in **(A)** is 30 s long. R, rest; LGO, imagined left hand gripping and opening; RGO, imagined right hand gripping and opening; SVM, support vector machine.

All participants underwent real-time fMRI scanning at the National Center for Geriatrics and Gerontology in Japan. The outline of the experimental protocol and tasks is given in Figure 1A. Each scanning session consisted of the following scans: (1) an anatomical localizer scan, (2) T1-weighted high resolution anatomical image scan, and (3) 4 motor imagery task fMRI scans (runs 0 to 3). All task fMRI scans consisted of nine rest (R) and eight task blocks with each block lasting for 30 s. The task blocks were divided into four blocks of imagined left hand gripping and opening (LGO) and four blocks of imagined right hand gripping and opening (RGO). In the first motor imagery scan (run 0), no feedback was provided, while for the remaining three scans (runs 1–3), feedback signals were given. In-between feedback scans, participants were given 5 min of rest.

Participants were also given time to practice the motor imagery tasks outside the scanner. During this practice session, the participants were initially asked to imagine gripping and opening their right or left hand to get a sense of the motor imagery tasks. This was done without time constraint. Once the participants felt comfortable doing the imagery tasks, they were then asked to actually perform the movements and instructed to remember the physical sensation of these movements as an additional guide for the imagery tasks. Finally, participants performed half of the task protocol corresponding to two blocks each of LGO and RGO tasks with rest in-between tasks using the same timing (30 s each block) and visual cue presentation in front of the stimulus computer. This was done to familiarize the participants with the actual tasks and timing during the scans. Participants were also instructed to continue doing the same imagery task even if the robot does not respond during the scans.

### Imaging Parameters

All scans were acquired using a Siemens Magnetom Trio (Siemens, Erlangen, Germany) 3.0 T MRI scanner with a 12-channel head coil. Anatomical T1-weighted MR images were acquired using a 3D MPRAGE (Magnetization Prepared Rapid Acquisition Gradient Echo, Siemens) pulse sequence (Mugler and Brookeman, 1990) with the following imaging parameters: repetition time (TR) = 2.53 s, echo time (TE) = 2.64 ms, 208 sagittal slices with a 50% distance factor and 1 mm thickness, field of view (FOV) = 250 mm, 256 × 256 matrix dimension and in plane voxel resolution of about 1.0 mm × 1.0 mm × 1.0 mm. The functional images were acquired using a gradient echo (GE) echo planar imaging (EPI) sequence with the following parameters: TR = 2.0 s, TE = 30 ms, flip angle (FA) = 80 degrees, 37 axial slices with a distance factor of 30% and thickness of 3.0 mm, FOV = 192 mm, 64 × 64 matrix dimension, voxel resolution = 3.0 mm × 3.0 mm × 3.0 mm, and a total of 255 volumes.

### Neurofeedback Training

For the feedback scans, we used our previously reported BMI system (Bagarinao et al., 2018). The system employed a small humanoid robot (KHR-3V, Kondo Science, Japan), the arms of which could be controlled (e.g., raising or lowering) via its USB connection to the real-time analysis system. During real-time fMRI scan, the system operates as follows. Each acquired image volume is immediately sent to the analysis system, which then processes the data. The preprocessed data is then fed into a previously trained SVM for real-time classification. If the target brain activation pattern has been identified, the analysis system will send a command (e.g., raise left arm) to the humanoid robot for immediate execution. The action of the robot is captured by a video camera, which sends the live video feed to the participant via a projector.

In this study, SVMs were trained to classify the different brain states associated with the imagery tasks and the trained SVMs were used to classify, in real-time, the ongoing brain state of the participants during feedback scans. Specifically, during task blocks, each acquired image volume was processed immediately by the analysis system, then classified by the trained SVM, and depending on the classification result, a command signal was sent to the humanoid robot. If SVM classified the volume as task (LGO/RGO) as compared to rest, the arm of the humanoid robot corresponding to the imagery task would be raised by 11 degrees, while for incorrect classification, the arm of the robot would remain stationary. Thus, during the task block, the robot’s arm would be continuously raised with an increment of 11 degrees depending on the SVM’s classification output. The higher the accuracy, the higher the corresponding arm would be raised. At the end of the task block, the arm would be reset to its initial downward position. During rest blocks, the robot stayed still and the participants focused their attention on a cross mark positioned on the robot’s body. The feedback was designed to be representative of or consistent with the task design. Since the classification was based on rest vs. task, when the classification was incorrect (SVM predicted rest during task blocks), the robot would not move, and when the classification was correct, the robot would move.

Unlike previous approaches, we used an incremental training strategy outlined in Figure 1B to train the SVMs during feedback scans. Specifically, the data from run 0 was used to train initial SVMs (SVM0) to classify rest from LGO (R vs. LGO) or rest from RGO (R vs. RGO) brain states. The trained SVMs were then used to classify in real time the acquired volumes obtained during feedback scan 1 (run 1). After the scan, the SVMs were updated and retrained using only the newly acquired data from run 1. The data from run 0 were not included in retraining the SVMs. The newly trained SVMs (SVM1) were then used to classify the data in the following feedback scan (run 2). This updating process was repeated until the last feedback scan.

For online and real-time image preprocessing, we used Statistical Parametric Mapping (SPM8, Wellcome Trust Center for Neuroimaging, London, United Kingdom) running on Matlab (R2016b, MathWorks, Natick, MA, United States). Data obtained during run 0 were immediately preprocessed after the scan (online preprocessing). The acquired functional images were realigned using a two-pass approach as implemented in SPM8. In the first pass, the images were co-registered to the first image in the series using a rigid body spatial transformation (three parameters for translation and three parameters for rotation) and the mean functional image, *I*_mean_, was then computed. In the second pass, the realigned images were further co-registered to the computed mean image. This mean image was also used as the reference image when aligning all functional images acquired during feedback scans. After realignment, the participant’s anatomical T1-weighted image was then co-registered to *I*_mean_ and segmented into component images including gray matter (GM), white matter (WM), and cerebrospinal fluid (CSF). The segmentation step also generated the transformation information from subject space to the Montreal Neurological Institute (MNI) template space. Using this information, the realigned functional images were normalized to MNI, resampled to a 3 mm × 3 mm × 3 mm voxel resolution and smoothed using an 8-mm full-width-at-half-maximum (FWHM) 3D Gaussian kernel. Finally, we applied a whole brain mask to the preprocessed images to exclude voxels outside the brain. This preprocessed data were then used to train SVM0. Although the normalization step is unnecessary for individual analysis, we performed this step so that results could be easily validated and compared with that of the other subjects. Moreover, we also used a common whole brain mask to limit SVM analysis to voxels within the brain and normalization made this easier. During feedback scans, each functional image was preprocessed immediately after acquisition (real-time preprocessing). The image was first realigned to *I*_mean_ estimated from run 0, then normalized to the MNI space, resampled to a 3 mm × 3 mm × 3 mm voxel resolution, smoothed using an 8-mm FWHM 3D Gaussian kernel, masked using the same whole brain mask employed in run 0 to exclude voxels outside the brain, incrementally detrended, and used as input to the trained SVMs for the real-time classification of the brain’s activation pattern. The SVM’s output was also detrended to correct for possible classifier drift (LaConte et al., 2007).

### Offline Data Analysis

We also performed offline analyses of the acquired data to generate the activation maps associated with the imagery tasks. For this, we used SPM12. The T1-weighted images were first segmented into component images including GM, WM, and CSF, among others, using SPM’s segmentation approach (Ashburner and Friston, 2005). The bias-corrected anatomical image as well as the transformation information from subject space to MNI space were then obtained. For the functional images, the first 5 volumes were discarded to account for the initial image inhomogeneity. The remaining images were then realigned relative to the mean functional image, co-registered to the bias-corrected anatomical image, normalized to the standard MNI space using the transformation information obtained from the segmentation step, resampled to a 2 mm × 2 mm × 2 mm voxel resolution, and spatially smoothed using an 8-mm FWHM 3D Gaussian filter.

Using the preprocessed functional images, the activation maps associated with all imagery tasks were generated for each participant. To do this, we used a box-car convolved with SPM’s canonical hemodynamic response function to model each task (LGO/RGO). The 6 estimated realignment parameters were also included as covariates of no interest to account for the effects of head motion. Contrast images were extracted for the LGO and RGO tasks as well as contrasts comparing the two tasks (LGO > RGO). One-sample *t*-tests of the resulting contrast maps were also performed to generate activation maps at the group level. All statistical maps were corrected for multiple comparisons at the cluster level with *p* < 0.05 using family-wise error correction (FWEc) and a cluster-defining threshold (CDT) set to *p* = 0.001 as implemented in SPM12. To test our hypothesis of the changing activation patterns during feedback scans, we performed a one-way repeated measures analysis of variance (ANOVA) using contrast images for each participants from feedback runs 1 to 3 for both LGO and RGO tasks. *Post hoc* paired sample *t*-tests were also performed between pairs of feedback runs. We used the Neuromorphometrics atlas available in SPM12 to label the different cortical areas in the obtained statistical maps. Surface projections of the activation maps are shown using BrainNet Viewer (Xia et al., 2013).

Offline SVM analyses were also performed using the preprocessed data and employing the same training strategy outlined in Figure 1B for more precise and detailed analyses. Three SVM classification models including R vs. LGO, R vs. RGO, and LGO vs. RGO were investigated. The third model was added to evaluate the discrimination of the representation of the imagery movement between the left and the right hands. Since feedback training only involved the first two models, the classification performance of the third model could serve as an indirect measure to quantify learning of the imagery tasks, that is, the higher the classification performance, the better the participants were able to generate distinct activation patterns for the two tasks in spite of the fact that the feedback was solely based on the classification of rest vs. task (LGO/RGO) brain states. To evaluate the SVMs’ performance for the first two classification models, we computed the task predictive value (TPV), defined as the ratio between the number of correctly classified task volumes and the total number of task volumes. Specifically, for R vs. LGO classification model, TPV was defined as the ratio between the number of correctly classified LGO volumes and the total number of LGO volumes within the run. The TPV for R vs. RGO classification model was defined in the same way. For the LGO vs. RGO classification model, we used accuracy defined as the total number of correctly classified LGO and RGO volumes over the total number of LGO and RGO volumes. Rest volumes were excluded in these definitions since rest blocks were not monitored during the scan and participants might also have practiced the task during rest blocks, which could lead to inaccurate classification. We also investigated the effect of SVM re-training strategy on the estimated classification measures using a one-way repeated-measures ANOVA. In all SVM analyses, we used a linear SVM and set the regularization parameter *c* to 1. We used the default value of *c* since this value has provided robust classification performance on the same classification problem based on our previous study (Bagarinao et al., 2018). All analyses were performed in Matlab (R2016b, MathWorks, Natick, MA, United States) using in-house scripts and LIBSVM (Chang and Lin, 2011), a free library for SVMs.

To identify regions that significantly contributed to the trained SVM’s classification performance, we performed a one-sample *t*-test to the weights obtained from all participants for each of the classification model above using SPM12. This is to test whether the mean weight value at each voxel across participants were significantly greater than or less than 0. The resulting statistical maps were then corrected for multiple comparisons at the cluster level using FWEc *p* < 0.05 (CDT *p* = 0.001) as implemented in SPM12.

### Validation Analyses

To further demonstrate the advantages of updating SVMs during a series of neurofeedback training runs, we performed additional offline validation analyses using the preprocessed data. The goal of these additional analyses was to contrast the classification performance when SVMs were only trained using data from run 0 and tested using data from feedback runs 1–3 with no training update, which is the typical approach. For these analyses, the same 3 SVM classification models (R vs. LGO, R vs. RGO, and LGO vs. RGO) were evaluated using the same performance measures (TPV for the first two models and accuracy for the third model).

## Results

### Improvement in Classification Performance With SVM Re-training

**FIGURE 2:**
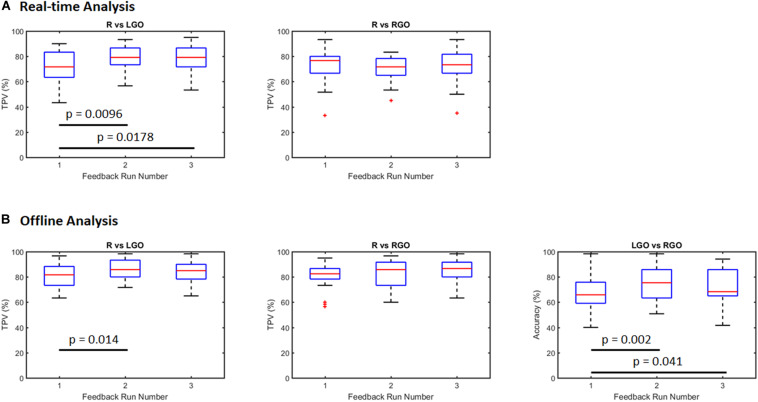
Classification performance for support vector machines (SVMs) trained using the incremental strategy outlined in Figure 1B **(A)** during real-time neurofeedback training (real-time analysis) and **(B)** offline analysis of the same data set. R, rest; LGO, imagined left hand gripping and opening; RGO, imagined right hand gripping and opening; TPV, task predictive value.

Figure 2 shows box plots of the classification performance of the SVMs trained using the incremental approach. Means and standard deviations are summarized in Table 1. For the real-time SVM performance (Figure 2A), the mean TPV values were 71.06, 78.83, and 78.28 for R vs. LGO and 72.78, 70.22, and 72.78 for R vs. RGO during feedback runs 1, 2, and 3, respectively. The result of one-way repeated-measures ANOVA showed significant main effect (*F*_2,58_ = 4.99, *p* = 0.010) in TPV values across feedback runs for R vs. LGO classification driven by the improvement in TPV between runs 1 and 2 (*p* = 0.0096, *post hoc* paired sample *t*-test) and runs 1 and 3 (*p* = 0.0178). For R vs. RGO classification, no significant (*F*_2,58_ = 0.536, *p* = 0.588) change was observed in TPV values across feedback runs. The list of TPV values for all participants is given in Supplementary Table S1.

**TABLE 1:** Support vector machine (SVM) classification performance for the different classification models.

	Real-time analysis		Offline analysis		
	R vs. LGO (TPV, %)	R vs. RGO (TPV, %)	R vs. LGO (TPV, %)	R vs. RGO (TPV, %)	LGO vs. RGO (Accuracy, %)
Run 1	71.06 (13.52)	72.78 (12.69)	81.00 (9.54)	81.00 (9.35)	67.17 (14.72)
Run 2	78.83 (11.16)	70.22 (09.30)	85.78 (8.11)	82.67 (10.34)	74.03 (13.53)
Run 3	78.28 (11.21)	72.78 (12.36)	84.17 (8.19)	85.61 (8.63)	72.06 (13.59)

*R, rest; LGO, imagined left hand gripping and opening; RGO, imagined right hand gripping and opening; TPV, task predictive value; numbers enclosed in parentheses represent standard deviation values.*

For the offline analysis (Figure 2B), we observed improvement in the classification performance as compared to that obtained during real-time analysis. This is mostly driven by better image preprocessing and additional optimization during offline analysis. The mean TPV values were 81.00, 85.78, and 84.17 for R vs. LGO classification and 81.00, 82.67, and 85.61 for R vs. RGO classification during feedback runs 1 to 3, respectively. Similarly, the mean accuracy values were 67.17, 74.03, and 72.06 for LGO vs. RGO classification. The results of the one-way repeated-measures ANOVA showed significant main effect in TPV values for R vs. LGO classification (*F*_2,58_ = 3.9107, *p* = 0.0255) and in accuracies for LGO vs. RGO classification (*F*_2,58_ = 5.2610, *p* = 0.0079) across feedback runs. Again, no significant change (*F*_2,58_ = 2.3711, *p* = 0.1024) was observed in TPV values for R vs. RGO classification. *Post hoc* paired sample *t*-tests showed significant improvement in TPV values between scans 1 and 2 for R vs. LGO classification (*p* = 0.014). The accuracy in classifying LGO vs. RGO also significantly improved between scans 1 and 2 (*p* = 0.002) as well as between scans 1 and 3 (*p* = 0.041). The list of TPV values and classification accuracies for all participants is given in Supplementary Table S2.

### SVM Performance in the Validation Analyses

**FIGURE 3:**
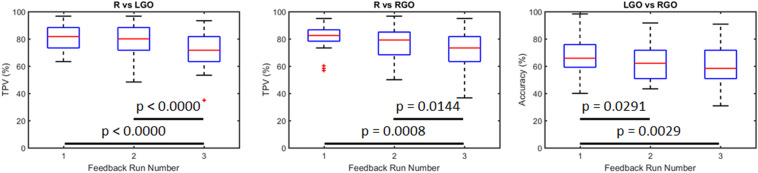
Classification performance for support vector machines trained using data from run 0 and tested using data from feedback runs 1 to 3 without training update. R, rest; LGO, imagined left hand gripping and opening; RGO, imagined right hand gripping and opening; TPV, task predictive value.

Figure 3 shows the SVM performance in the validation analyses of the preprocessed data. Using only the data from run 0 to train the SVMs and testing the trained SVM on data from runs 1 to 3, the mean TPV values for the R vs. LGO classification for runs 1 to 3 were 81.00, 78.00, and 71.44, respectively. For the R vs. RGO classification task, the mean TPV values for runs 1 to 3 were 81.00, 76.89, and 71.72, respectively, and for the LGO vs. RGO classification, the mean accuracies were 67.17, 63.44, and 60.56, respectively. One-way repeated measures ANOVA showed significant main effect in TPV values for R vs. LGO classification (*F*_2,58_ = 18.4031, *p* < 0.0000) and for R vs. RGO classification (*F*_2,58_ = 9.0382, *p* = 0.0004) as well as in the classification accuracy for LGO vs. RGO classification (*F*_2,58_ = 7.0906, *p* = 0.0018) across feedback runs. *Post hoc* paired sample *t*-tests showed significant decrease in TPV values between runs 1 and 3 (*p* < 0.0000 for R vs. LGO and *p* = 0.0008 for R vs. RGO) and runs 2 and 3 (*p* < 0.0000 for R vs. LGO and *p* = 0.0144 for R vs. RGO) as well as in the classification accuracy between runs 1 and 2 (*p* = 0.0291) and runs 1 and 3 (*p* = 0.0029) for the LGO vs. RGO classification model. These results suggest that without re-training, the SVM performance significantly decreases with each feedback run. The list of TPV values and accuracies for all participants is given in Supplementary Table S3.

### Changes in Activation Patterns During Feedback Runs

**FIGURE 4:**
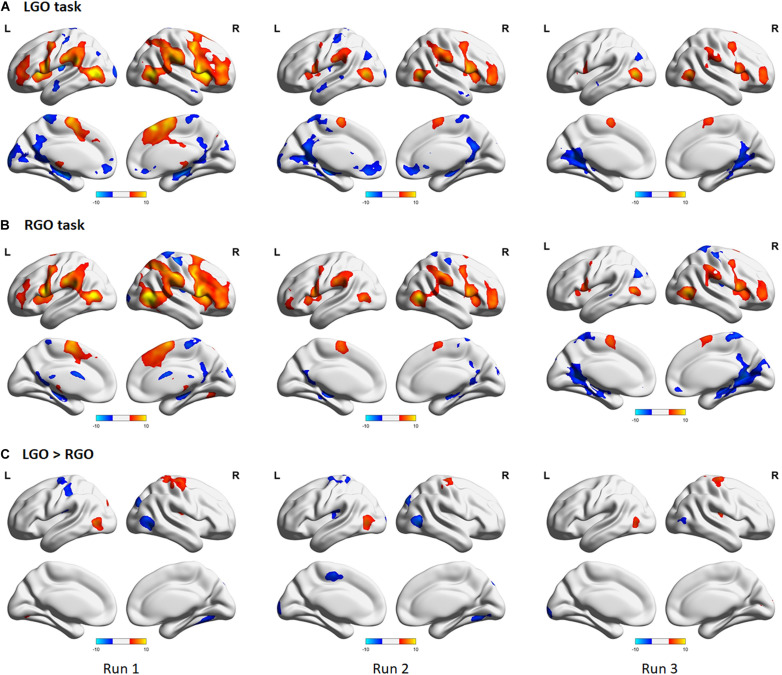
Group activation maps for imagined **(A)** left hand gripping and opening (LGO) and **(B)** right hand gripping and opening (RGO) tasks and **(C)** the contrast (LGO > RGO) between the two tasks obtained using a one-sample *t*-test from individual contrast maps for feedback runs 1 to 3 (left to right). All statistical maps were corrected for multiple comparisons at the cluster level using family-wise error correction (FWEc) *p* < 0.05 with a cluster-defining threshold (CDT) set at *p* = 0.001.

Figure 4 shows the group-level activation maps for the different tasks during feedback runs 1 to 3 generated using one-sample *t*-tests of the corresponding subject-level activation maps. Based on this figure, we could clearly see that the activation pattern changed from feedback run 1 to run 3 with the former being more widespread and the latter more focused on relevant brain regions. Clusters showing significant activation/deactivation during feedback runs 1 to 3 are summarized in Tables 2–4, respectively.

**TABLE 2:** Clusters showing significant (FWEc *p* < 0.05) activation and deactivation during feedback run 1 for the different motor imagery tasks.

	*x*	*y*	*z*	*z-*val	Cluster size	Area	Other peaks
**LGO**							
Activation	−52	−68	6	7.555	5064	L MTG	L SMG
	32	20	4	7.534	20006	R AIns	R SMC, R CO
	52	−60	2	6.797	7118	R MTG	R SMG
	−36	16	4	6.696	5616	L FO	L PrG
	−24	−64	−24	6.670	2537	L Cer	
	28	−62	−24	5.271	418	R Cer	
Deactivation	−38	−16	20	7.162	493	L CO	
	−30	−8	−20	5.700	8038	L Hip	
	−50	−22	58	5.551	476	L PoG	
	−10	−40	70	5.550	933	L PoG	R MPrG, R PoG
	−4	56	−8	4.954	1123	L MFC	L MSFG, R MFC
	−36	−78	42	4.374	244	L AnG	
**RGO**							
Activation	52	−62	2	Inf	10032	R MTG	R SMG, R Cer
	−46	−66	8	7.195	5273	L MTG	L SMG, L PO
	50	8	14	6.807	23271	R OpIFG	R SMC, L PrG
	−28	−64	−24	6.262	2116	L Cer	R Cer
Deactivation	40	−14	22	6.022	5995	R CO	R Hip
	40	−24	62	5.931	1828	R PoG	R MPrG
	22	−92	2	5.086	752	R OCP	L Cun
**LGO vs. RGO**							
LGO > RGO	−52	−72	2	5.377	1110	L IOG	L MTG
	44	−14	20	5.071	496	R CO	R Pu
	30	−18	62	4.784	1863	R PrG	
	−18	−86	32	4.203	238	L SOG	L MOG
LGO < RGO	24	−52	−18	5.074	2114	R Cer	R ITG, R IOG
	−50	−24	18	4.698	294	L PO	
	24	−82	32	4.615	284	R SOG	
	−42	−28	42	4.590	1120	L PoG	L PrG

**LGO, imagined left hand gripping and opening; RGO, imagined right hand gripping and opening; MTG, middle temporal gyrus; SMG, supramarginal gyrus; AIns, anterior insula; SMC, supplementary motor cortex; CO, central operculum; FO, frontal operculum; PrG, precentral gyrus; Cer, cerebellum; Hip, hippocampus; PoG, postcentral gyrus; MPrG, medial precentral gyrus; MFC, medial frontal cortex; MSFG, medial superior frontal gyrus; AnG, angular gyrus; OpIFG, opercular part of the inferior frontal gyrus; OCP, occipital pole; Cun, cuneus; IOG, inferior occipital gyrus; Pu, putamen; SOG, superior occipital gyrus; MOG, middle occipital gyrus; ITG, inferior temporal gyrus; PO, posterior operculum; L, left; R, right.**

**TABLE 3:** Clusters showing significant (FWEc *p* < 0.05) activation and deactivation during feedback run 2 for the different motor imagery tasks.

	*x*	*y*	*z*	*z*-val	Cluster size	Area	Other peaks
**LGO**							
Activation	46	−64	4	6.287	1015	R IOG	
	−46	−70	4	6.195	1038	L IOG	
	56	12	12	6.162	3477	R OpIFG	R AIns
	60	−32	44	5.581	2625	R SMG	
	46	42	−2	5.570	1176	R TrIFG	R LOrG, R MFG
	−46	0	6	5.509	1396	L CO	L OpIFG, L AIns
	−6	−4	68	5.453	972	L SMC	R SMC, R SFG
	−60	−24	26	5.434	1068	L SMG	
	−30	−68	−22	4.361	531	L Cer	
	18	−8	8	4.099	556	R ThP	R Pallidum
Deactivation	−36	−16	22	5.794	850	L CO	L PCgG, L MCgG
	−34	−26	−14	5.773	5710	L Hip	L OCP, L PCu
	−2	38	−14	5.099	1603	L ACgG	R SCA, R ACgG
	36	−42	−8	4.838	829	R FuG	R Hip
	−18	−40	60	4.791	1946	L PoG	
	−38	−76	40	4.614	602	L AnG	
	58	4	−14	4.055	279	R STG	R TMP, R MTG
**RGO**							
Activation	46	−66	4	6.761	4579	R IOG	R SMG
	52	10	14	6.023	3523	R OpIFG	R PrG
	−50	4	14	5.717	2265	L PrG	L AIns, L CO
	−66	−26	34	5.496	1544	L SMG	L PO
	42	46	−4	5.290	1591	R MFG	
	−46	−62	2	5.185	656	L MTG	
	−36	42	−16	5.051	526	L LOrG	L OrIFG, L MFG
	−6	−2	58	4.969	1133	L SMC	R SMC
	26	−62	−24	4.705	614	R Cer	
	−26	−64	−24	4.307	284	L Cer	
	−20	−4	14	3.830	332	L Cau	L ThP
Deactivation	−36	−44	−8	5.058	933	L FuG	L Hip, L PHG
	12	−40	74	4.611	397	R PoG	R SPL, R MPoG
	26	−18	−14	4.577	569	R Hip	
	44	−20	58	4.451	445	R PoG	R PrG
	−8	−50	6	4.226	1188	L PCgG	R PCu, L PCu
**LGO vs. RGO**							
LGO > RGO	−46	−72	2	5.193	649	L IOG	
	32	−18	58	4.798	636	R PrG	R PoG
LGO < RGO	24	−86	38	5.525	378	R SOG	R MOG
	46	−64	2	5.395	1988	R IOG	R Cer
	−22	−14	74	5.047	1217	L PrG	L SPL, L SFG
	−44	−26	22	4.696	931	L PO	L Pu
	−8	−16	54	4.543	378	L SMC	L MPrG
	−12	−100	8	4.008	350	L OCP	L Calc

**LGO, imagined left hand gripping and opening; RGO, imagined right hand gripping and opening; IOG, inferior occipital gyrus; OpIFG, opercular part of the inferior frontal gyrus; AIns, anterior insula; SMG, supramarginal gyrus; TrIFG, triangular part of the inferior frontal gyrus; LOrG, lateral orbital gyrus; MFG, middle frontal gyrus; CO, central operculum; SMC, supplementary motor cortex; SFG, superior frontal gyrus; Cer, cerebellum; ThP, thalamus proper; PCgG, posterior cingulate gyrus; MCgG, midcingulate gyrus; Hip, hippocampus; OCP, occipital pole; PCu, precuneus; ACgG, anterior cingulate gyrus; SCA, subcallosal area; FuG, fusiform gyrus; PoG, postcentral gyrus; AnG, angular gyrus; STG, superior temporal gyrus; TMP, temporal pole; MTG, middle temporal gyrus; PrG, precentral gyrus; PO, posterior operculum; OrIFG, orbital part of the inferior frontal gyrus; Cau, caudate; PHG, parahippocampal gyrus; SPL, superior parietal lobule; MPoG, medial postcentral gyrus; SOG, superior occipital gyrus; MOG, middle occipital gyrus; Pu, putamen; MPrG, medial precentral gyrus; Calc, calcarine; L, left; R, right*.*

**TABLE 4:** Clusters showing significant (FWEc *p* < 0.05) activation and deactivation during feedback run 3 for the different motor imagery tasks.

	*x*	*y*	*z*	*z*-val	Cluster size	Area	Other peaks
**LGO**							
Activation	−48	−70	4	6.411	785	L IOG	
	50	8	12	5.567	2445	R OpIFG	R FO, R PrG
	46	−64	4	5.494	799	R IOG	
	−28	−64	−24	5.028	564	L Cer	
	−6	−4	66	4.879	825	L SMC	R SMC, R SFG
	40	48	8	4.814	775	R MFG	
	−22	−72	−44	4.611	313	L Cer	
	−54	4	14	4.555	420	L PrG	L FO, L AIns
	60	−22	26	4.452	1119	R SMG	
Deactivation	−12	−48	0	4.791	5004	L PCgG	R PCgG, L PCu
	−42	−72	30	4.109	378	L AnG	
**RGO**							
Activation	52	−62	0	5.956	1218	R MTG	R IOG
	48	8	12	5.906	2939	R OpIFG	R PrG, R FO
	−48	−68	6	5.420	405	L MTG	
	−20	−72	−44	4.973	531	L Cer	
	42	48	0	4.918	1056	R MFG	R LOrG
	58	−32	42	4.858	1743	R SMG	R STG
	−52	4	14	4.843	1269	L PrG	L CO
	−6	−2	58	4.755	1015	L SMC	R SMC
	−18	−10	18	4.243	318	L ThP	L Cau
Deactivation	18	−50	8	5.907	9945	R PCu	R PoG
	34	−12	22	5.859	358	R CO	
	−40	−70	32	4.901	828	L AnG	L SOG
	4	48	−14	4.138	399	R MFC	L SFG, L MSFG
**LGO vs. RGO**							
LGO > RGO	36	−14	20	5.504	369	R CO	R PIns, R Pu
	24	−18	74	5.190	1114	R PrG	
	−50	−70	4	4.593	463	L IOG	L MTG
	18	−94	6	4.569	310	R OCP	R Calc
	−24	−38	−24	4.051	399	L Cer	
LGO < RGO	−12	−98	−6	4.670	187	L Calc	
	42	−72	8	4.132	206	R IOG	

**LGO, imagined left hand gripping and opening; RGO, imagined right hand gripping and opening; IOG, inferior occipital gyrus; OpIFG, opercular part of the inferior frontal gyrus; FO, frontal operculum; PrG, precentral gyrus; Cer, cerebellum; SMC, supplementary motor cortex; SFG, superior frontal gyrus; MFG, middle frontal gyrus; AIns, anterior insula; SMG, supramarginal gyrus; PCgG, posterior cingulate gyrus; PCu, precuneus; AnG, angular gyrus; MTG, middle temporal gyrus; LOrG, lateral orbital gyrus; STG, superior temporal gyrus; CO, central operculum; ThP, thalamus proper; Cau, caudate; PoG, postcentral gyrus; SOG, superior occipital gyrus; MFC, medial frontal cortex; MSFG, medial superior frontal gyrus; PIns, posterior insula; Pu, putamen; OCP, occipital pole; Calc, calcarine; L, left; R, right.**

For the LGO task (Figure 4A), regions that were consistently activated across feedback runs included the bilateral inferior occipital gyrus/middle temporal gyrus, right opercular part of the inferior frontal gyrus/anterior insula, left cerebellum (not shown in the figure), bilateral supplementary motor cortex/right superior frontal gyrus, right middle frontal gyrus, left precentral gyrus/anterior insula/frontal and central operculum, and right supramarginal gyrus. Regions activated only during feedback runs 1 and 2 included the left supramarginal gyrus and right thalamus proper/pallidum. The left cerebellum (inferior portion, not shown) was activated during feedback runs 1 and 3, while the right cerebellum (not shown) was activated only during feedback run 1. On the other hand, consistent deactivations across feedback runs were observed in the bilateral posterior cingulate gyrus and the left angular gyrus. The left central operculum, left hippocampus, bilateral anterior cingulate gyrus/medial frontal cortex, right fusiform gyrus/hippocampus, and the left postcentral gyrus/medial precentral gyrus were deactivated during feedback runs 1 and 2, while the right superior temporal gyrus/temporal pole/middle temporal gyrus was deactivated during feedback run 2.

For the RGO task (Figure 4B), regions that were consistently activated across feedback runs included the right middle temporal gyrus/inferior occipital gyrus, right opercular part of the inferior frontal gyrus/precentral gyrus, left middle temporal gyrus, left cerebellum (not shown), right middle frontal gyrus, right supramarginal gyrus, left precentral gyrus, bilateral supplementary motor cortex, and the left thalamus proper. The left supramarginal gyrus and the right cerebellum were activated only during feedback runs 1 and 2, while the left lateral part of the orbital gyrus was activated only during feedback run 2. On the other hand, only the right postcentral gyrus was consistently deactivated across feedback runs, while the bilateral precuneus/posterior cingulate gyrus and the left fusiform gyrus/parahippocampal gyrus were deactivated during feedback runs 2 and 3, the right central operculum during feedback runs 1 and 3, the left angular gyrus and right medial frontal cortex during feedback run 3, and the right occipital pole during feedback run 1.

Contrasting the two tasks (Figure 4C), regions showing consistent higher activations in the LGO task compared to the RGO task across all feedback runs included the right precentral gyrus and the left inferior occipital gyrus/middle temporal gyrus. The right central operculum showed higher activation in the LGO task compared to the RGO task only during feedback runs 1 and 3, while the left superior occipital gyrus showed higher activation during feedback run 1, and the right occipital pole and left cerebellum only during feedback run 3. In contrast, only the right inferior occipital gyrus showed consistent higher activation in the RGO task compared to the LGO task across all feedback runs. The right superior occipital gyrus, left precentral gyrus, left posterior operculum, and the right cerebellum also showed higher activation in the RGO task during feedback runs 1 and 2, while the left calcarine showed this during runs 2 and 3. Finally, the left supplementary motor cortex was higher in RGO task during feedback run 2 and the left postcentral gyrus during feedback run 1.

**FIGURE 5:**
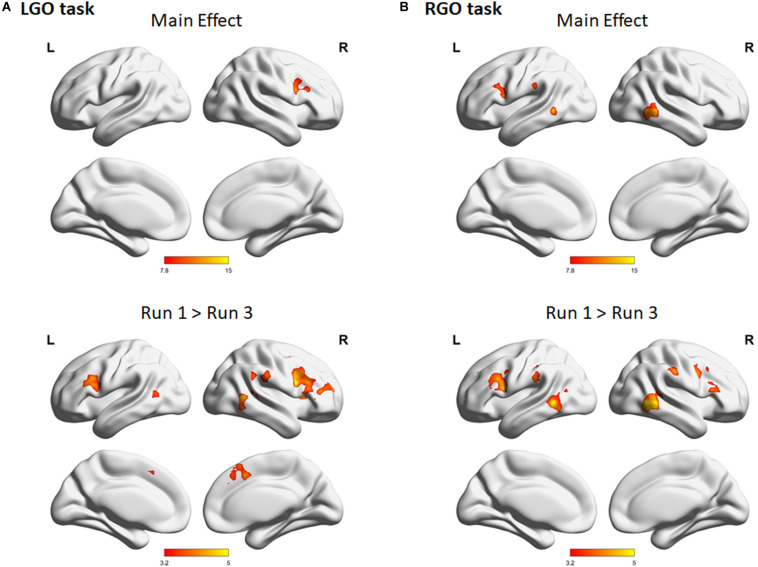
Regions showing significant main effect in one-way repeated measures analysis of variance for the imagined **(A)** LGO and **(B)** RGO tasks. Results of *post hoc* paired sample *t*-tests for both LGO and RGO tasks are also shown in the bottom figures. All statistical maps were corrected for multiple comparisons using FWEc *p* < 0.05 with CDT *p* = 0.001.

The results of the one-way repeated-measures ANOVA showed significant main effect for both tasks across feedback runs (Figure 5). For the LGO task (Figure 5A), significant changes in activation can be seen in the right middle frontal gyrus/opercular part of the inferior frontal gyrus, while for the RGO task (Figure 5B), in the bilateral middle temporal gyrus, left supramarginal gyrus, and left precentral gyrus. These changes were mostly driven by a decrease in activation in these regions between feedback runs 1 and 3 (Figure 5, lower figures). Aside from these regions, other regions showing significant decrease in activation included the bilateral middle temporal gyrus, right supplementary motor cortex, left middle frontal gyrus, and right supramarginal gyrus for the LGO task and the right opercular part of the inferior frontal gyrus/precentral gyrus and right supramarginal gyrus for the RGO task. The list of clusters showing significant changes across feedback runs, including peak MNI coordinates and cluster sizes, is given in Table 5.

**TABLE 5:** Clusters showing significant main effect in a one-way repeated measures analysis of variance for the different tasks across feedback runs.

	*x*	*y*	*z*	*z*-val	Cluster size	Area	Other peaks
**LGO**							
Main	38	22	18	4.839	888	R MFG	R OpIFG
Run 1 > run 3	38	22	18	5.176	3115	R MFG	R OpIFG, R SFG
	52	−54	6	4.487	704	R MTG	R ITG
	10	16	44	4.300	793	R SMC	R SFG
	−42	−58	8	4.216	606	L MTG	
	−48	28	24	4.165	1089	L MFG	L OpIFG
	60	−26	50	4.075	474	R SMG	
**RGO**							
Main	58	−46	−6	4.494	524	R MTG	
	−66	−26	30	4.377	259	L SMG	
	−52	6	16	4.357	517	L PrG	L OpIFG, L TrIFG
	−62	−50	−2	4.330	229	L MTG	
Run 1 > run 3	58	−46	−6	4.918	1047	R MTG	
	−62	−50	−2	4.839	1958	L MTG	L SMG
	58	24	18	4.701	1197	R OpIFG	R PrG
	−60	14	22	4.629	1194	L OpIFG	L PrG, L TrIFG
	54	−22	34	4.414	379	R SMG	

**LGO, imagined left hand gripping and opening; RGO, imagined right hand gripping and opening; MFG, middle frontal gyrus; OpIFG, opercular part of the inferior frontal gyrus; SFG, superior frontal gyrus; MTG, middle temporal gyrus; ITG, inferior temporal gyrus; SMC, supplementary motor cortex; SMG, supramarginal gyrus; PrG, precentral gyrus; TrIFG, triangular part of the inferior frontal gyrus; L, left; R, right.**

### Significant SVM Weights

**FIGURE 6:**
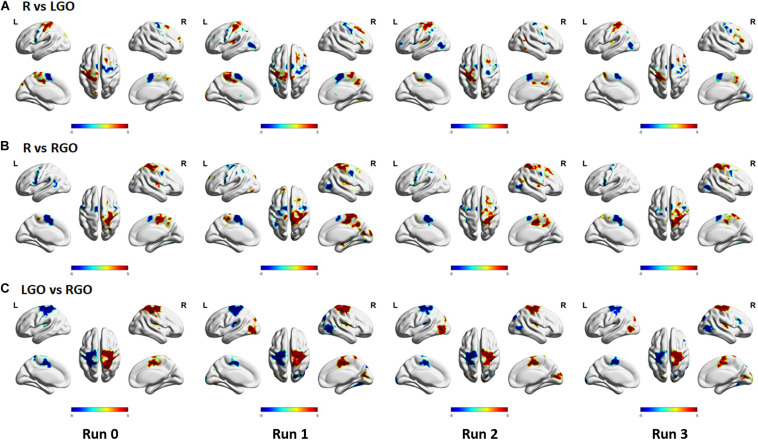
Significant weights (FWEc *p* < 0.05 with CDT *p* = 0.001) of the different SVM classification models obtained using a one-sample *t*-test of the SVM weights from all participants: **(A)** R vs. LGO, **(B)** R vs. RGO, and **(C)** LGO vs. RGO. Red–yellow color map indicates that the activity during rest (R) is higher compared to that during task (LGO/RGO) in R vs. LGO and R vs. RGO models and higher during LGO task compared to that during RGO task in LGO vs. RGO model. Blue–light blue color map indicates the opposite.

Support vector machines weights of the three classification models that are significant are shown in Figure 6. Regions that had generally higher activity during rest as compared to that during task (LGO/RGO) are shown in red-yellow colors in Figures 6A,B, or during LGO task as compared to RGO task in Figure 6C, whereas the opposite condition is shown in blue – light blue colors. Most of the relevant regions in the sensorimotor network appeared to have significant weights across runs. An important difference is the absence of the middle temporal gyrus/inferior occipital gyrus in the significance maps for run 0 as compared to that for runs 1–3. This difference is mainly due to the absence of feedback in run 0. Moreover, in spite of the decreasing activation intensity difference between LGO and RGO tasks across feedback runs (Figure 4C), regions in the sensorimotor network consistently showed significant weight values across feedback runs. The total number of voxels showing significant weight values for the 3 classification models also decreased from run 1 to run 3, although this is not so apparent in the figure. The lists of clusters with significant (FWEc *p* < 0.05, CDT *p* = 0.001) SVM weights for the 3 classification models are given in Supplementary Tables S4–S6.

## Discussion

Using a motor imagery paradigm, we investigated the effect of the dynamic changes in brain activation during neurofeedback training to the classification of the different brain states associated with the target tasks. These changes could be driven by learning effects and possibly other factors. Our findings confirmed our hypothesis that brain activation patterns could dynamically change as the training progresses. By continuously adapting the trained SVMs after every feedback runs, significant improvement in the classification of the different brain states associated with the target motor imagery tasks could be attained. For BMI applications, this improvement could lead to better control of the system. And for neurofeedback training, this could provide more reliable feedback information to the participants, which is necessary to attain a successful neurofeedback training.

The goal of neurofeedback training is for participants to learn the target tasks using feedback information directly derived from their ongoing brain activity. This entails that dynamic changes in brain activation could potentially occur as the training progressed. Based on our findings, we did observe a more widespread activation during feedback run 1 followed by a more focused and localized patterns in succeeding runs. This initial widespread activity could be driven by the recruitment of additional brain regions needed to learn the target new skill. As the participants acquired the needed skill, irrelevant regions would no longer be activated whereas the relevant ones would become more prominent. This similar trend was also observed for connectivity patterns during neurofeedback training where connectivity was initially widespread among several regions and later confined to a small number of regions after participants presumably consolidated regulation skills (Rota et al., 2011). Thus, these activation changes should be considered when identifying different brain states during neurofeedback training.

Previous neurofeedback studies using brain state classification used datasets acquired before training to train classifiers, which would then be employed to classify the different brain states in succeeding feedback scans (see approach in validation analyses). Many of these studies had observed that the performance of classifiers trained this way usually decreased across feedback runs. LaConte (2011) attributed this effect to learning, suggesting that activation patterns changed during feedback training when participants learned the target tasks. Using a simple motor learning task, they demonstrated that the classification accuracy of the trained SVM did decrease when participants learned the task, whereas for those who did not, the accuracy remained the same or even increased. In our previous study (Bagarinao et al., 2018), we also observed the same trend, although the observed decrease was not statistically significant across the three feedback runs. To further support these previous findings, we performed the validation analyses (see section “Validation Analyses”) in which we trained SVMs using data from run 0 and used the trained SVMs to classify data from runs 1 to 3. These supplemental analyses were primarily performed to demonstrate that without re-training, the classifiers’ performance would be significantly affected due to the changing activation pattern (Figure 4). As is clearly evident in Figure 3, the classifiers’ performance did decrease with each feedback run. Using a very limited number of participants (2 participants), Sitaram et al. (2011) had also demonstrated that participants learned to improve their performance (measured in terms of classification accuracy) using an incremental method of re-training the classifiers. Our findings showing increases in TPV and accuracy values with continuous SVM re-training further validated their result for an extended number of participants.

One could also argue that even without SVM re-training, the classification performance could still be improved when participants learned the task since activation regions were becoming more well-defined (Figure 4). However, SVMs trained using data with a more widespread activation pattern could assign significant weights to regions that were initially active but not really relevant to the task. Without SVM re-training, these regions would still contribute to the classification even if these regions were no longer active after learning, thus affecting the SVM’s performance as clearly demonstrated in the results of the validation analyses (Figure 3). With SVM re-training, regions irrelevant to the task could be assigned non-significant weights to improve performance. Indeed, as shown in Figure 6 and Supplementary Tables S4–S6, the number of voxels with significant weights decreased with each feedback run, suggesting that some regions weighted as significant in the previous runs were no longer significant in feedback run 3. Although the presence of other confounding factors (e.g., learning) during neurofeedback training could also influence the SVM’s performance, the results reasonably indicated that the observed improvement in TPV values and accuracies was most likely driven by SVM re-training.

A consequence of the incremental training approach is the possibility that with training, the learned activation may not converge in the direction of the preferred activation pattern. This could be driven, for example, by the participants trying to explore different strategies for the target task, then the SVMs adjusting to the change in activation patterns and providing updated feedback to the participants, which in turn, affects the participants’ choice of strategy. This “adaptive” training is an intriguing scenario, but is unlikely with the current protocol. Here, we used two predefined tasks where the activation patterns are known in advance. Moreover, participants were also instructed to continue doing the same task even if there was no response from the robot (no movement). To identify if indeed the training results are consistent with the training goals, one can examine the resulting activation map, which can be readily generated when using real-time fMRI as compared to other modalities. Alternatively, one can also include a control task from which the target task can be contrasted or compared with, as performed in this study. This is particularly useful to detect cases where participants just perform something to contrast with the rest condition. Lastly, one can also include behavioral measures that can be evaluated for a successful training. Adaptive training is an interesting problem that will need more detailed investigation possibly using a different task design and is beyond the scope of the current paper.

Although the primary focus of the paper is to examine the efficacy of incremental training to improve SVMs’ classification performance when activation patterns dynamically change during training, our findings have also shown indications of the possible effects of the improvement in the classification to the participants’ ability to learn the task-relevant activation patterns. In our previous study (Bagarinao et al., 2018) comparing participants’ performance of motor imagery tasks with and without feedback, we have shown that participants were able to generate more consistent brain activation patterns that are relevant to the tasks during sessions with neurofeedback as compared to that without feedback, suggesting the importance of the feedback information. In that study, the SVMs were only trained using data from run 0 (the one without feedback) and the trained SVMs were subsequently used in the succeeding feedback runs. Under this condition, we found no significant improvement in the classification performance across feedback runs and the SVMs’ performance even showed some tendency to decrease with each feedback run. In terms of activation, we also did not observed significant changes across feedback runs. In contrast to these findings, we have demonstrated here that with incremental SVM training, improvement in accuracy could be observed across feedback runs. This improvement was also accompanied by significant changes in brain activation in the direction that lead to more focal activations in task-relevant brain regions. Although the way the feedback was presented differed in these two studies, and thus limiting a direct comparison, the motor imagery tasks were the same. Taken together, the findings of these two studies seemed to suggest the possibility that the improvement in SVMs’ performance has provided participants with better feedback information, which in turn, has led them to generate the more focal and task-relevant activation patterns. To fully address this association, more detailed investigations such as having a direct control group will be necessary.

In terms of activations, we identified several regions activated during feedback runs. Some of these were consistent with regions usually associated with motor imagery tasks including supplementary motor cortex, premotor cortex, prefrontal cortex, posterior parietal cortex, cerebellum, and basal ganglia (O’Shea and Moran, 2017; Tong et al., 2017). Interestingly, we did not find activations in the contralateral primary motor cortex (M1), an important target region for neurorehabilitation applications, for both imagery tasks at the group level. Instead, the ipsilateral M1 was consistently deactivated in all feedback runs for the RGO task and in the first two runs for the LGO task (Figure 4), consistent with previous results (Chiew et al., 2012). Findings concerning M1 activation during motor imagery task are inconsistent. A meta-analysis based on 75 papers showed that only 22 out of the 122 experiments reported M1 activation (Hétu et al., 2013). In a recent study using fMRI-based neurofeedback, Mehler et al. (2019) also reported that M1 could not be activated during imagery task in spite of the provided feedback information, supporting our finding. In contrast, they reported consistent activation of the supplementary motor cortex, which is also similar to what we observed.

The activation of other regions could be related to processes associated with neurofeedback training. In a recent review, Sitaram et al. (2017) had proposed three neurofeedback-related networks that included the dorsolateral prefrontal cortex, thalamus, lateral occipital, and posterior parietal cortex as regions for the control of visual neurofeedback, the dorsal striatum for neurofeedback learning, and the ventral striatum, anterior cingulate cortex, and anterior insula for neurofeedback reward. In this study, we also observed similar activation for some of these regions. For instance, we observed consistent activation of the left/right inferior occipital gyrus/middle temporal gyrus, regions implicated for the control of visual neurofeedback. These regions were also observed to be active when feedback was provided during motor imagery task but not without feedback (Bagarinao et al., 2018). It is also interesting to note that some of the regions associated with neurofeedback-related processes overlap with that of the motor imagery task (e.g., posterior parietal, anterior insula, and others). This could be due to the fact that neurofeedback training also involved some form of imagery.

Comparing across feedback runs, we observed that some regions showed significant decrease in activation with training. One such region is the inferior occipital gyrus/middle temporal gyrus. This region has been implicated in several neurofeedback studies (Berman et al., 2012; Marchesotti et al., 2017; Bagarinao et al., 2018) and, as mentioned earlier, could be associated with the control of visual feedback (Sitaram et al., 2017). The observed decreased activity of this region with training could indicate that participants were slowly decreasing their reliance on the feedback information as they learned the imagery tasks. Similar decreases in activation were also observed in the contralateral middle frontal gyrus/opercular part of the inferior frontal gyrus. Higher activation of this region has been associated with early learning or with a still incomplete motor sequence acquisition (Müller et al., 2002). Like the inferior occipital gyrus, this region may also be involved in the initial learning of the motor imagery tasks and becomes less activated as the new skill is acquired.

We also observed significant improvement in LGO task classification across feedback runs as compared to RGO task (Figure 2). Since most participants were right handed, this may be associated with the influence of handedness in learning motor imagery. An earlier study has demonstrated that motor imagery abilities are unbalanced between dominant and non-dominant hands (Maruff et al., 1999) with the dominant hand showing better performance (Guillot et al., 2010; Paizis et al., 2014). This behavioral difference could produce asymmetrical brain activation. Based on a magnetoencephalography study, Boe et al. (2014) have shown that the non-dominant hand induced a stronger event-related desynchronization in the ipsilateral sensorimotor cortex than in the contralateral cortex. This greater activation was considered as an indication of the control group’s inability to perform the motor imagery task with the non-dominant hand. Our data also showed consistent deactivation during RGO (dominant) task in the ipsilateral sensorimotor region, but not during the LGO (non-dominant) task (Figure 4). These differences in activation may explain why the LGO task showed significant improvement in classification across feedback runs than the RGO task. In this case, motor imagery with the non-dominant hand exhibited stronger and more dynamic activation pattern during training, leading to classification improvements whereas that of the dominant hand appeared more stable, resulting in more consistent classification.

Finally, we note the relevance of our findings for motor imagery training, particularly in relation to its clinical applications. Several studies have examined the application of motor imagery training as a no-cost, safe, and easy way to enhance motor functions. Motor imagery training has been employed to improve athletes’ performance (Feltz and Landers, 1983), provide additional benefits to conventional physiotherapy (Zimmermann-Schlatter et al., 2008), and enhance motor recovery following stroke (Jackson et al., 2001; Sharma et al., 2006; de Vries and Mulder, 2007). Motor imagery can also be used to identify potential sources of residual functional impairment in well-recovered stroke patients (Sharma et al., 2009a, b). By providing neurofeedback, motor imagery training has been shown to be more effective for neurorehabilitation. For example, adding BMI system in conjunction with motor imagery training to provide online contingent sensory feedback of brain activity has been shown effective in improving clinical parameters of post-stroke motor recovery (Ramos-Murguialday et al., 2013; Ono et al., 2014; Frolov et al., 2017). In healthy participants, using neurofeedback has been shown to significantly improve volitional recall of motor imagery activation patterns (Bagarinao et al., 2018). In these approaches, the BMI systems actively decode the brain activity and display the outcome to the user to create a feedback that is reflective of the task performance. Pilot studies using real-time fMRI-based neurofeedback for motor function recovery in stroke patients have also shown some promise (Sitaram et al., 2012; Liew et al., 2016). Using motor-imagery-based strategies, patients were able to increase connectivity between cortical and subcortical regions (Liew et al., 2016) and increase regulation of the activity in regions relevant to motor function (Sitaram et al., 2012; Lioi et al., 2020). An adaptive, multi-target motor imagery training approach was also proposed by Lioi et al. (2020). They used two target regions where the regions’ contributions to the feedback signal were weighted and the weight values were adjusted at the latter part of the training. In principle, this is similar to the proposed incremental SVM training approach. With SVM, regions are weighted according to their contribution to the classification. With the proposed incremental training, the weights can be adjusted according to the changes in activation pattern as the training progresses. Thus, further optimizing neurofeedback-based motor imagery training using the incremental strategy could be beneficial in improving the reliability of feedback information during motor imagery task training. This approach could also be used to customize differences in learning strategies, which may vary among individuals.

One of the limitations of the current study is the small number of feedback runs to continuously assess the improvement in accuracy with SVM re-training. We used only a limited number of runs to minimize task fatigue, which would increase with more feedback runs and could also introduce changes in activation pattern. Another limitation is the lack of independent instruments to assess the improvement in motor imagery performance of the participants during or after training. In the absence of such instruments, we used an indirect measure based on the accuracy of classifying the LGO task against the RGO task. Since the feedback information was mainly based on rest vs. task (LGO/RGO) performance, improvement in LGO vs. RGO classification would suggest better separation of the tasks’ activation patterns, which could be taken as an indication of the improvement in motor imagery performance in both tasks.

## Conclusion

Our results confirmed our hypothesis that activation patterns could dynamically change during neurofeedback training as participants learned to perform the target motor imagery tasks. To account for these changes, we employed a training strategy that continuously updates the trained classifiers after every feedback run, resulting in the improvement of the SVM’s overall classification performance. This is important in order to provide more reliable and accurate feedback information to participants during neurofeedback training, an essential factor that could affect the effectiveness of neurofeedback in clinical settings.

## Data Availability Statement

The datasets generated for this study will not be made publicly available due to privacy and legal reasons. Requests to access these datasets should be referred to TN at nakai.toshiharu@nitech.ac.jp.

## Ethics Statement

This study was reviewed and approved by the Institutional Review Board of the National Center for Geriatrics and Gerontology of Japan. All participants provided written informed consent before joining the study.

## Author Contributions

EB, AY, SK, and TN conceived and designed the study. KT and SK assembled and programmed the humanoid robot for real-time fMRI application. EB developed the real-time fMRI analysis system. EB, AY, KT, and TN performed the experiments and analyzed the data. TN gave technical support and supervised the whole research procedure. EB and TN wrote the draft of the manuscript. All authors reviewed and approved the final version of the manuscript.

## Conflict of Interest

The authors declare that the research was conducted in the absence of any commercial or financial relationships that could be construed as a potential conflict of interest.
