# Supplementary material for: Improving Real-Time Brain State Classification of Motor Imagery Tasks During Neurofeedback Training
Source: Front Neurosci. 2020 Jun 24;14:623. doi: 10.3389/fnins.2020.00623 (PMC7326956; doi:10.3389/fnins.2020.00623)
Supplement: Supplementary file 1 [file Table_1.docx]

Supplementary Material

# Supplementary Tables

**Supplementary Table S1**. Classification performance of the trained support vector machines used during real-time neurofeedback training for all participants

|  | R vs LGO (TPV, %) | | | R vs RGO (TPV, %) | | |
| --- | --- | --- | --- | --- | --- | --- |
| ID | **Run 1** | **Run 2** | **Run 3** | **Run 1** | **Run 2** | **Run 3** |
| s001 | 46.67 | 86.67 | 75.00 | 33.33 | 70.00 | 35.00 |
| s002 | 70.00 | 91.67 | 71.67 | 85.00 | 75.00 | 85.00 |
| s003 | 46.67 | 68.33 | 65.00 | 93.33 | 78.33 | 70.00 |
| s004 | 86.67 | 86.67 | 93.33 | 51.67 | 80.00 | 93.33 |
| s005 | 71.67 | 81.67 | 80.00 | 68.33 | 73.33 | 76.67 |
| s006 | 66.67 | 83.33 | 76.67 | 53.33 | 61.67 | 75.00 |
| s007 | 76.67 | 56.67 | 61.67 | 76.67 | 66.67 | 78.33 |
| s008 | 71.67 | 73.33 | 73.33 | 75.00 | 68.33 | 63.33 |
| s009 | 78.33 | 83.33 | 78.33 | 66.67 | 81.67 | 61.67 |
| s010 | 53.33 | 76.67 | 65.00 | 51.67 | 83.33 | 63.33 |
| s011 | 43.33 | 70.00 | 83.33 | 63.33 | 80.00 | 81.67 |
| s012 | 81.67 | 76.67 | 86.67 | 88.33 | 66.67 | 81.67 |
| s013 | 51.67 | 56.67 | 71.67 | 80.00 | 45.00 | 73.33 |
| s014 | 66.67 | 76.67 | 75.00 | 65.00 | 75.00 | 58.33 |
| s015 | 61.67 | 78.33 | 93.33 | 78.33 | 71.67 | 78.33 |
| s016 | 83.33 | 85.00 | 65.00 | 70.00 | 71.67 | 50.00 |
| s017 | 63.33 | 80.00 | 91.67 | 78.33 | 65.00 | 88.33 |
| s018 | 88.33 | 78.33 | 53.33 | 71.67 | 76.67 | 61.67 |
| s019 | 73.33 | 63.33 | 91.67 | 65.00 | 53.33 | 83.33 |
| s020 | 81.67 | 75.00 | 95.00 | 73.33 | 71.67 | 70.00 |
| s021 | 65.00 | 63.33 | 58.33 | 80.00 | 61.67 | 70.00 |
| s022 | 85.00 | 91.67 | 86.67 | 85.00 | 83.33 | 71.67 |
| s023 | 85.00 | 91.67 | 81.67 | 83.33 | 63.33 | 73.33 |
| s024 | 83.33 | 90.00 | 80.00 | 81.67 | 71.67 | 86.67 |
| s025 | 68.33 | 93.33 | 85.00 | 76.67 | 78.33 | 73.33 |
| s026 | 60.00 | 93.33 | 75.00 | 71.67 | 70.00 | 90.00 |
| s027 | 90.00 | 58.33 | 88.33 | 78.33 | 56.67 | 66.67 |
| s028 | 75.00 | 86.67 | 93.33 | 76.67 | 78.33 | 83.33 |
| s029 | 66.67 | 76.67 | 73.33 | 76.67 | 56.67 | 71.67 |
| s030 | 90.00 | 91.67 | 80.00 | 85.00 | 71.67 | 68.33 |
| MEAN | **71.06** | **78.83** | **78.28** | **72.78** | **70.22** | **72.78** |

Abbreviations: R, rest; LGO, imagined left hand gripping and opening; RGO, imagined right hand gripping and opening; TPV, task predictive value

**Supplementary Table S2**. Classification performance of the trained support vector machines in the offline analyses of the data for all participants

|  | R vs LGO (TPV, %) | | | R vs RGO (TPV, %) | | | LGO vs RGO (accuracy, %) | | |
| --- | --- | --- | --- | --- | --- | --- | --- | --- | --- |
| ID | **Run 1** | **Run 2** | **Run 3** | **Run 1** | **Run 2** | **Run 3** | **Run 1** | **Run 2** | **Run 3** |
| s001 | 76.67 | 83.33 | 86.67 | 78.33 | 73.33 | 86.67 | 60.83 | 72.50 | 55.83 |
| s002 | 78.33 | 93.33 | 80.00 | 86.67 | 66.67 | 91.67 | 52.50 | 60.83 | 75.00 |
| s003 | 71.67 | 83.33 | 81.67 | 88.33 | 85.00 | 71.67 | 61.67 | 73.33 | 70.83 |
| s004 | 88.33 | 93.33 | 96.67 | 86.67 | 96.67 | 93.33 | 65.83 | 78.33 | 89.17 |
| s005 | 81.67 | 95.00 | 81.67 | 78.33 | 91.67 | 91.67 | 70.83 | 91.67 | 80.83 |
| s006 | 80.00 | 80.00 | 83.33 | 60.00 | 88.33 | 75.00 | 56.67 | 55.00 | 52.50 |
| s007 | 88.33 | 75.00 | 76.67 | 78.33 | 76.67 | 85.00 | 71.67 | 50.83 | 66.67 |
| s008 | 75.00 | 85.00 | 81.67 | 81.67 | 95.00 | 86.67 | 72.50 | 85.83 | 83.33 |
| s009 | 95.00 | 85.00 | 83.33 | 86.67 | 91.67 | 80.00 | 61.67 | 64.17 | 65.83 |
| s010 | 81.67 | 88.33 | 88.33 | 73.33 | 66.67 | 80.00 | 40.00 | 70.83 | 58.33 |
| s011 | 63.33 | 91.67 | 90.00 | 58.33 | 91.67 | 90.00 | 80.83 | 88.33 | 85.83 |
| s012 | 83.33 | 93.33 | 88.33 | 91.67 | 91.67 | 86.67 | 80.83 | 77.50 | 76.67 |
| s013 | 66.67 | 78.33 | 75.00 | 88.33 | 68.33 | 83.33 | 57.50 | 55.00 | 70.00 |
| s014 | 73.33 | 73.33 | 73.33 | 56.67 | 76.67 | 63.33 | 49.17 | 77.50 | 67.50 |
| s015 | 73.33 | 80.00 | 90.00 | 78.33 | 81.67 | 90.00 | 62.50 | 64.17 | 65.00 |
| s016 | 96.67 | 93.33 | 98.33 | 81.67 | 91.67 | 83.33 | 98.33 | 92.50 | 86.67 |
| s017 | 73.33 | 86.67 | 91.67 | 90.00 | 85.00 | 90.00 | 42.50 | 63.33 | 67.50 |
| s018 | 85.00 | 98.33 | 78.33 | 86.67 | 90.00 | 96.67 | 81.67 | 86.67 | 89.17 |
| s019 | 80.00 | 71.67 | 83.33 | 73.33 | 85.00 | 90.00 | 42.50 | 51.67 | 63.33 |
| s020 | 90.00 | 75.00 | 71.67 | 83.33 | 73.33 | 66.67 | 59.17 | 61.67 | 57.50 |
| s021 | 65.00 | 80.00 | 65.00 | 88.33 | 66.67 | 76.67 | 61.67 | 70.00 | 66.67 |
| s022 | 81.67 | 91.67 | 88.33 | 80.00 | 91.67 | 90.00 | 75.83 | 89.17 | 94.17 |
| s023 | 95.00 | 86.67 | 78.33 | 90.00 | 86.67 | 86.67 | 75.00 | 79.17 | 41.67 |
| s024 | 90.00 | 95.00 | 88.33 | 85.00 | 70.00 | 95.00 | 95.00 | 95.00 | 88.33 |
| s025 | 91.67 | 85.00 | 96.67 | 85.00 | 90.00 | 83.33 | 86.67 | 77.50 | 94.17 |
| s026 | 85.00 | 98.33 | 88.33 | 83.33 | 91.67 | 96.67 | 65.83 | 83.33 | 69.17 |
| s027 | 76.67 | 73.33 | 70.00 | 81.67 | 60.00 | 78.33 | 70.83 | 60.83 | 65.83 |
| s028 | 83.33 | 91.67 | 93.33 | 80.00 | 86.67 | 91.67 | 65.83 | 80.83 | 57.50 |
| s029 | 65.00 | 75.00 | 86.67 | 75.00 | 78.33 | 90.00 | 61.67 | 65.00 | 65.83 |
| s030 | 95.00 | 93.33 | 90.00 | 95.00 | 91.67 | 98.33 | 87.50 | 98.33 | 90.83 |
| MEAN | **81.00** | **85.78** | **84.17** | **81.00** | **82.67** | **85.61** | **67.17** | **74.03** | **72.06** |

Abbreviations: R, rest; LGO, imagined left hand gripping and opening; RGO, imagined right hand gripping and opening; TPV, task predictive value

**Supplementary Table S3**. Classification performance of the support vector machines trained using run 0 data and tested using data from runs 1 to 3 for all participants in the validation analyses

|  | R vs LGO (TPV, %) | | | R vs RGO (TPV, %) | | | LGO vs RGO (accuracy, %) | | |
| --- | --- | --- | --- | --- | --- | --- | --- | --- | --- |
| ID | **Run 1** | **Run 2** | **Run 3** | **Run 1** | **Run 2** | **Run 3** | **Run 1** | **Run 2** | **Run 3** |
| s001 | 76.67 | 78.33 | 71.67 | 78.33 | 80.00 | 70.00 | 60.83 | 64.17 | 60.00 |
| s002 | 78.33 | 83.33 | 65.00 | 86.67 | 66.67 | 85.00 | 52.50 | 64.17 | 71.67 |
| s003 | 71.67 | 75.00 | 83.33 | 88.33 | 93.33 | 70.00 | 61.67 | 69.17 | 63.33 |
| s004 | 88.33 | 85.00 | 81.67 | 86.67 | 88.33 | 71.67 | 65.83 | 60.83 | 65.83 |
| s005 | 81.67 | 83.33 | 83.33 | 78.33 | 81.67 | 81.67 | 70.83 | 58.33 | 59.17 |
| s006 | 80.00 | 73.33 | 70.00 | 60.00 | 68.33 | 38.33 | 56.67 | 46.67 | 35.83 |
| s007 | 88.33 | 75.00 | 68.33 | 78.33 | 63.33 | 61.67 | 71.67 | 50.83 | 50.83 |
| s008 | 75.00 | 78.33 | 80.00 | 81.67 | 80.00 | 71.67 | 72.50 | 83.33 | 75.83 |
| s009 | 95.00 | 93.33 | 81.67 | 86.67 | 96.67 | 80.00 | 61.67 | 70.00 | 55.00 |
| s010 | 81.67 | 71.67 | 65.00 | 73.33 | 83.33 | 85.00 | 40.00 | 50.83 | 52.50 |
| s011 | 63.33 | 73.33 | 63.33 | 58.33 | 66.67 | 63.33 | 80.83 | 76.67 | 80.83 |
| s012 | 83.33 | 91.67 | 71.67 | 91.67 | 73.33 | 53.33 | 80.83 | 70.83 | 60.83 |
| s013 | 66.67 | 58.33 | 60.00 | 88.33 | 65.00 | 63.33 | 57.50 | 43.33 | 50.83 |
| s014 | 73.33 | 61.67 | 60.00 | 56.67 | 53.33 | 60.00 | 49.17 | 43.33 | 57.50 |
| s015 | 73.33 | 48.33 | 35.00 | 78.33 | 50.00 | 36.67 | 62.50 | 55.00 | 40.83 |
| s016 | 96.67 | 90.00 | 93.33 | 81.67 | 88.33 | 85.00 | 98.33 | 91.67 | 90.83 |
| s017 | 73.33 | 83.33 | 76.67 | 90.00 | 78.33 | 63.33 | 42.50 | 47.50 | 30.83 |
| s018 | 85.00 | 95.00 | 83.33 | 86.67 | 95.00 | 95.00 | 81.67 | 71.67 | 90.00 |
| s019 | 80.00 | 65.00 | 55.00 | 73.33 | 68.33 | 76.67 | 42.50 | 50.83 | 51.67 |
| s020 | 90.00 | 75.00 | 70.00 | 83.33 | 63.33 | 76.67 | 59.17 | 47.50 | 48.33 |
| s021 | 65.00 | 58.33 | 53.33 | 88.33 | 70.00 | 66.67 | 61.67 | 48.33 | 48.33 |
| s022 | 81.67 | 91.67 | 80.00 | 80.00 | 85.00 | 81.67 | 75.83 | 78.33 | 74.17 |
| s023 | 95.00 | 88.33 | 91.67 | 90.00 | 81.67 | 70.00 | 75.00 | 60.83 | 62.50 |
| s024 | 90.00 | 83.33 | 83.33 | 85.00 | 76.67 | 65.00 | 95.00 | 86.67 | 88.33 |
| s025 | 91.67 | 81.67 | 68.33 | 85.00 | 88.33 | 75.00 | 86.67 | 79.17 | 70.83 |
| s026 | 85.00 | 88.33 | 75.00 | 83.33 | 86.67 | 80.00 | 65.83 | 62.50 | 52.50 |
| s027 | 76.67 | 61.67 | 60.00 | 81.67 | 71.67 | 75.00 | 70.83 | 61.67 | 50.83 |
| s028 | 83.33 | 90.00 | 78.33 | 80.00 | 75.00 | 78.33 | 65.83 | 56.67 | 46.67 |
| s029 | 65.00 | 61.67 | 53.33 | 75.00 | 85.00 | 81.67 | 61.67 | 62.50 | 45.00 |
| s030 | 95.00 | 96.67 | 81.67 | 95.00 | 83.33 | 90.00 | 87.50 | 90.00 | 85.00 |
| MEAN | **81.00** | **78.00** | **71.44** | **81.00** | **76.89** | **71.72** | **67.17** | **63.44** | **60.56** |

Abbreviations: R, rest; LGO, imagined left hand gripping and opening; RGO, imagined right hand gripping and opening; TPV, task predictive value

**Supplementary Table S4**. Clusters with significant (FWEc p < 0.05, CDT p = 0.001) SVM weights for the R vs LGO classification model

|  | x | y | z | zval | cluster size | area | other peaks |
| --- | --- | --- | --- | --- | --- | --- | --- |
| Run 0 | | | | | | | |
| Positive | -40 | -18 | 20 | 5.53 | 192 | L CO |  |
|  | -28 | -28 | 72 | 5.47 | 3113 | L PoG | L MPrG |
|  | 20 | 16 | 60 | 4.89 | 233 | R SFG |  |
|  | -14 | -80 | 38 | 4.53 | 166 | L Cun | L SOG |
|  | 28 | 56 | 14 | 4.19 | 133 | R MFG | R SFG |
| Negative | 2 | 0 | 56 | 6.95 | 2309 | R SMC | L SFG, R PrG |
|  | 46 | 4 | 6 | 5.52 | 151 | R CO |  |
|  | -40 | 10 | 60 | 5.41 | 146 | L MFG |  |
|  | 26 | -4 | 4 | 5.15 | 303 | R Pu | R Cau |
|  | -22 | -62 | -18 | 4.67 | 400 | L Cer | L FuG, L LiG |
|  | -28 | -60 | -58 | 4.56 | 304 | L Cer |  |
|  | -54 | 2 | 44 | 4.30 | 100 | L PrG |  |
|  | -56 | 6 | 18 | 4.10 | 111 | L PrG |  |
|  | 56 | 6 | 44 | 3.63 | 101 | R PrG |  |
| Run 1 | | | | | | | |
| Positive | -6 | -34 | 56 | 6.30 | 4354 | L MPrG | L PoG |
|  | 26 | 26 | 52 | 5.88 | 509 | R SFG | R MFG |
|  | -36 | -22 | 6 | 5.54 | 522 | L TTG | L CO |
|  | 50 | 30 | 12 | 4.75 | 105 | R TrIFG |  |
|  | 22 | -8 | -16 | 4.63 | 96 | R Amyg | R Hip |
|  | -16 | -96 | 0 | 4.59 | 216 | L OCP | L OFuG |
|  | 24 | -62 | -10 | 4.08 | 113 | R LiG |  |
| Negative | -4 | 4 | 56 | 6.49 | 1532 | L SMC | R SMC |
|  | -52 | -72 | 4 | 5.91 | 696 | L IOG |  |
|  | -56 | 8 | 20 | 5.48 | 100 | L PrG |  |
|  | 24 | -2 | 4 | 5.24 | 692 | R Pu | R Cau |
|  | -38 | 58 | 20 | 5.24 | 378 | L MFG |  |
|  | 50 | 2 | 42 | 5.14 | 1272 | R PrG |  |
|  | -44 | -64 | 50 | 4.94 | 344 | L AnG | L SPL |
|  | -56 | 2 | 38 | 4.62 | 166 | L PrG |  |
|  | -8 | 4 | 2 | 4.44 | 267 | L Cau | L Pu, L AIns |
|  | -18 | -54 | -22 | 4.34 | 128 | L Cer |  |
|  | -18 | -64 | -50 | 4.11 | 98 | L Cer |  |
| Run 2 | | | | | | | |
| Positive | -34 | -38 | 62 | 5.55 | 1990 | L PoG |  |
|  | -8 | -30 | 48 | 5.44 | 491 | L MPrG | L MCgG, R SMC |
|  | -44 | -22 | 20 | 5.37 | 425 | L PO | L PIns |
|  | 4 | -50 | 36 | 4.95 | 377 | R PCu | R PCgG |
|  | 20 | 12 | 56 | 4.68 | 228 | R SFG | R MFG |
|  | 40 | -64 | 20 | 4.46 | 302 | R MOG | R AnG |
|  | 20 | -88 | 34 | 4.08 | 113 | R SOG |  |
|  | 58 | -48 | -10 | 4.04 | 110 | R MTG | R ITG |
| Negative | -4 | -2 | 60 | 6.28 | 2094 | L SMC | R PrG, R SMC |
|  | -46 | -72 | 2 | 6.27 | 521 | L IOG |  |
|  | -22 | -56 | -20 | 5.91 | 341 | L Cer |  |
|  | 22 | -2 | 6 | 5.68 | 516 | R Pu | R Cau |
|  | 50 | 4 | 4 | 5.64 | 250 | R CO |  |
|  | -52 | 6 | 0 | 5.46 | 177 | L CO |  |
|  | -44 | -52 | 54 | 5.16 | 569 | L AnG | L SMG, L SPL |
|  | -34 | 4 | 64 | 4.99 | 400 | L MFG | L PrG |
|  | -24 | 0 | 4 | 4.72 | 431 | L Pu | L Cau |
|  | -32 | 48 | 38 | 4.33 | 113 | L MFG |  |
|  | -42 | 50 | 6 | 4.12 | 132 | L MFG |  |
| Run 3 | | | | | | | |
| Positive | -4 | -42 | 68 | 6.07 | 2666 | L MPoG | L PoG, R PCu |
|  | 32 | 8 | 36 | 5.11 | 422 | R MFG |  |
|  | 46 | 30 | 18 | 5.10 | 298 | R MFG |  |
|  | -36 | -16 | 2 | 4.36 | 301 | L PIns | L CO |
|  | 22 | -42 | -22 | 4.03 | 151 | R Cer |  |
| Negative | -6 | -6 | 64 | 6.12 | 1100 | L SMC | R SMC |
|  | 36 | -14 | 54 | 6.03 | 727 | R PrG |  |
|  | -46 | -72 | 6 | 5.31 | 501 | L IOG | L MTG |
|  | 50 | 2 | 6 | 5.22 | 211 | R CO |  |
|  | 24 | -2 | 10 | 4.82 | 142 | R Pu |  |
|  | -44 | -70 | 48 | 4.60 | 103 | L AnG |  |
|  | -20 | -66 | -16 | 4.43 | 167 | L Cer | L LiG |
|  | -22 | 0 | 8 | 4.27 | 127 | L Pu | L Pal |
|  | -54 | 0 | 50 | 4.15 | 145 | L PrG | L MFG |
|  | 14 | -88 | 2 | 4.04 | 206 | R Calc |  |

* List of abbreviations below

**Supplementary Table S5**. Clusters with significant (FWEc p < 0.05, CDT p = 0.001) SVM weights for the R vs RGO classification model

|  | x | y | z | zval | cluster size | area | other peaks |
| --- | --- | --- | --- | --- | --- | --- | --- |
| Run 0 | | | | | | | |
| Positive | 28 | -42 | 68 | 6.29 | 3493 | R SPL | R PoG, R MPrG |
|  | 6 | -50 | 40 | 5.05 | 260 | R PCu | R SPL |
|  | 36 | -14 | 18 | 4.81 | 422 | R PIns | R CO, R PO |
|  | 20 | -76 | 30 | 4.60 | 93 | R Cun |  |
|  | 30 | -58 | 34 | 4.39 | 237 | R SPL |  |
|  | 28 | 26 | 52 | 4.02 | 144 | R MFG | R SFG |
|  | -18 | -92 | -8 | 3.85 | 125 | L OFuG | L OCP |
| Negative | -4 | 0 | 56 | 6.92 | 1454 | L SMC | L SFG, R SMC |
|  | -56 | 4 | 30 | 5.92 | 627 | L PrG |  |
|  | 32 | -48 | -30 | 5.48 | 407 | R Cer |  |
|  | -40 | -58 | 16 | 5.24 | 363 | L AnG | L MTG |
|  | -22 | -8 | 2 | 4.86 | 220 | L Pallidum | L Pu |
|  | 54 | 8 | 46 | 4.45 | 379 | R PrG |  |
|  | -58 | -34 | 24 | 4.44 | 108 | L PO | L PT |
|  | 24 | -62 | -52 | 4.39 | 139 | R Cer |  |
|  | -40 | 10 | 62 | 4.18 | 152 | L MFG | L SFG |
|  | -40 | -50 | 64 | 4.10 | 237 | L SPL | L AnG |
| Run 1 | | | | | | | |
| Positive | 0 | -28 | 60 | 6.32 | 5388 | L MPrG | R PrG, R PoG |
|  | 4 | -94 | 12 | 5.42 | 274 | L Cun | R Cun |
|  | 40 | -14 | -28 | 5.39 | 507 | R FuG | R Hip, R PIns |
|  | 38 | -10 | 14 | 4.95 | 555 | R PIns | R CO |
|  | 8 | -30 | 10 | 4.68 | 206 | R ThP |  |
|  | -18 | -88 | 34 | 4.62 | 148 | L SOG | L SPL |
|  | -18 | 54 | 32 | 4.56 | 161 | L SFG |  |
|  | 28 | 22 | 46 | 4.52 | 246 | R MFG | R SFG |
|  | -38 | -68 | -12 | 4.18 | 169 | L OFuG | L IOG |
| Negative | -4 | -6 | 58 | 6.53 | 1781 | L SMC |  |
|  | -56 | 10 | 20 | 5.53 | 337 | L OpIFG | L PrG |
|  | -40 | -52 | 46 | 5.49 | 302 | L AnG | L SPL |
|  | 48 | 2 | 44 | 5.34 | 706 | R PrG |  |
|  | 42 | -62 | 4 | 5.30 | 697 | R IOG |  |
|  | -24 | 8 | 0 | 5.28 | 517 | L Pu | L Cau |
|  | 32 | -44 | -30 | 5.13 | 903 | R Cer | R LiG |
|  | 22 | 12 | 2 | 5.04 | 253 | R Pu | R Cau, R ThP |
|  | -44 | -32 | 34 | 4.77 | 144 | L SMG | L PoG |
|  | 18 | -68 | -50 | 4.13 | 129 | R Cer |  |
| Run 2 | | | | | | | |
| Positive | 32 | -36 | 64 | 6.15 | 3531 | R PoG | R MPrG |
|  | 40 | -16 | 18 | 5.02 | 371 | R CO | R PIns |
|  | 24 | 26 | 44 | 4.81 | 620 | R MFG | R SFG |
|  | 56 | -46 | 26 | 4.33 | 146 | R AnG |  |
|  | -20 | -86 | 36 | 4.22 | 168 | R SOG |  |
| Negative | -2 | -4 | 56 | 6.48 | 1096 | L SMC | L SFG |
|  | -24 | 0 | 10 | 5.84 | 557 | L Pu | L ThP |
|  | 26 | 2 | 10 | 5.65 | 208 | R Pu |  |
|  | -50 | 8 | 0 | 5.34 | 304 | L FO | L AIns, L CO |
|  | 46 | -60 | 4 | 5.18 | 451 | R MTG | R IOG |
|  | 28 | -64 | -22 | 5.03 | 560 | R Cer | R LiG |
|  | -36 | -10 | 54 | 4.53 | 288 | L PrG | L SFG |
|  | 50 | -4 | 44 | 4.44 | 402 | R PrG |  |
|  | -46 | -38 | 50 | 4.41 | 222 | L SMG | L AnG |
|  | -54 | 4 | 32 | 4.30 | 258 | L PrG | L OpIFG |
|  | 62 | 6 | 28 | 4.11 | 106 | R PrG |  |
| Run 3 | | | | | | | |
| Positive | 12 | -44 | 70 | 5.63 | 3279 | R PoG |  |
|  | -22 | -44 | -24 | 4.57 | 103 | L Cer | L FuG |
|  | 54 | -8 | 14 | 4.49 | 119 | R CO | R PIns |
|  | 58 | -10 | 42 | 4.37 | 134 | R PoG |  |
|  | 42 | -6 | -12 | 4.28 | 105 | R PP |  |
|  | 32 | 10 | 54 | 3.99 | 98 | R MFG |  |
| Negative | -2 | -4 | 60 | 6.63 | 1239 | L SMC |  |
|  | -58 | 6 | 18 | 5.42 | 282 | L PrG | L CO, L FO |
|  | 44 | 4 | 58 | 5.13 | 461 | R MFG | R PrG |
|  | -16 | 0 | 8 | 5.10 | 411 | L Cau | L Pal, L Pu |
|  | 28 | -56 | -20 | 4.45 | 187 | R Cer |  |
|  | 44 | -70 | 0 | 4.41 | 347 | R IOG |  |
|  | -24 | -16 | 76 | 4.32 | 188 | L PrG |  |
|  | -10 | -82 | -14 | 4.24 | 144 | L LiG | L Calc |
|  | -32 | -6 | 58 | 4.06 | 158 | L PrG | L SFG |

* List of abbreviations below

**Supplementary Table S6**. Clusters with significant (FWEc p < 0.05, CDT p = 0.001) SVM weights for the LGO vs RGO classification model

|  | x | y | z | zval | cluster size | area | other peaks |
| --- | --- | --- | --- | --- | --- | --- | --- |
| Run 0 | | | | | | | |
| Positive | 28 | -12 | 64 | 7.27 | 5821 | R PrG |  |
|  | -16 | -60 | -48 | 5.72 | 468 | L Cer |  |
|  | -6 | -58 | -8 | 5.34 | 840 | L LiG | L Cer |
|  | 28 | -10 | 4 | 5.29 | 339 | R Pu |  |
|  | 56 | -22 | 16 | 4.60 | 633 | R PO | R CO, R PoG |
|  | 44 | 2 | 12 | 3.99 | 134 | R CO |  |
| Negative | -24 | -16 | 72 | 6.26 | 4684 | L PrG | L PoG, L SPL |
|  | -18 | -24 | 12 | 5.74 | 227 | L ThP |  |
|  | 22 | -50 | -18 | 5.58 | 641 | R Cer | R FuG |
|  | 14 | -60 | -50 | 5.36 | 659 | R Cer |  |
|  | -42 | -20 | 18 | 5.06 | 246 | L CO | L PIns |
| Run 1 | | | | | | | |
| Positive | 44 | -16 | 20 | 6.79 | 1497 | R CO |  |
|  | 24 | -10 | 66 | 6.70 | 5564 | R PrG |  |
|  | -12 | -54 | -14 | 5.90 | 2365 | L Cer | L IOG |
|  | 10 | -76 | 16 | 4.81 | 291 | R Calc | R LiG |
|  | -18 | -88 | 30 | 4.42 | 372 | L SOG |  |
|  | -22 | -58 | -54 | 4.31 | 191 | L Cer |  |
|  | 20 | -106 | 8 | 4.06 | 107 | R OCP |  |
| Negative | -26 | -14 | 70 | 6.54 | 4290 | L PrG | L PoG |
|  | 26 | -48 | -20 | 6.35 | 1441 | R Cer |  |
|  | -6 | -6 | 48 | 6.25 | 389 | L SMC | L MPrG |
|  | 8 | -64 | -46 | 5.84 | 443 | R Cer |  |
|  | 42 | -62 | 8 | 5.81 | 1946 | R MTG | R SOG, R IOG |
|  | -52 | -22 | 20 | 5.10 | 495 | L CO | L PIns |
|  | -8 | -100 | 6 | 4.29 | 119 | L OCP |  |
| Run 2 | | | | | | | |
| Positive | 26 | -22 | 70 | 6.71 | 4303 | R PrG |  |
|  | -42 | -70 | 4 | 5.37 | 1379 | L IOG |  |
|  | 38 | -16 | 18 | 5.37 | 649 | R CO | R PIns |
|  | -18 | -54 | -18 | 5.11 | 681 | L Cer |  |
|  | -20 | -60 | -56 | 4.62 | 208 | L Cer |  |
|  | 8 | -96 | 20 | 4.56 | 576 | R Cun | R Calc |
|  | -20 | -84 | 30 | 4.28 | 272 | L SOG |  |
| Negative | -22 | -8 | 72 | 6.70 | 3698 | L SFG | L PrG, L PoG |
|  | -42 | -22 | 20 | 5.54 | 294 | L PO | L CO |
|  | 26 | -50 | -28 | 5.40 | 725 | R Cer |  |
|  | 12 | -64 | -48 | 5.16 | 541 | R Cer |  |
|  | -14 | -98 | -6 | 4.73 | 344 | L OCP |  |
|  | 24 | -84 | 36 | 4.63 | 415 | R SOG |  |
|  | 56 | -74 | -6 | 4.28 | 567 | R IOG | R MTG |
| Run 3 | | | | | | | |
| Positive | 30 | -22 | 66 | 6.07 | 3940 | R PrG |  |
|  | -44 | -70 | 2 | 5.62 | 865 | L IOG |  |
|  | 42 | -16 | 16 | 5.54 | 631 | R CO | R PIns, R PO |
|  | -12 | -50 | -16 | 5.42 | 745 | L Cer |  |
|  | 10 | -76 | 8 | 4.72 | 654 | R Calc |  |
|  | -28 | -52 | -52 | 4.46 | 198 | L Cer |  |
|  | -20 | -80 | 26 | 4.20 | 351 | L SOG | L MOG |
| Negative | -4 | -8 | 48 | 6.14 | 3439 | L SMC | L PrG |
|  | 26 | -56 | -18 | 5.35 | 1717 | R Cer | R IOG |
|  | 54 | 26 | 36 | 4.79 | 349 | R MFG | R TrIFG |
|  | 22 | -84 | 38 | 4.68 | 281 | R SOG |  |
|  | -46 | -24 | 18 | 4.52 | 235 | L PO | L TTG, L CO |
|  | -12 | -98 | -2 | 4.28 | 208 | L Calc |  |
|  | 24 | -58 | -54 | 4.19 | 127 | R Cer |  |

* List of abbreviations below

**LIST OF ABBREVIATIONS**

AIns – anterior insula

Amyg – amygdala

AnG – angular gyrus

Calc – calcarine

Cau – caudate

CDT – cluster defining threshold

Cer – cerebellum

CO – central operculum

Cun – cuneus

FO – frontal operculum

FuG – fusiform gyrus

FWEc – family-wise error correction at the cluster level

Hip – hippocampus

IOG – inferior occipital gyrus

ITG – inferior temporal gyrus

L – left

LiG – lingual gyrus

MCgG – midcingulate gyrus

MFG – middle frontal gyrus

MOG – middle occipital gyrus

MPoG – medial postcentral gyrus

MPrG – medial precentral gyrus

MTG – middle temporal gyrus

OCP – occipital pole

OFuG – occipital fusiform gyrus

OpIFG – opercular part of the inferior frontal gyrus

Pal – pallidum

PCgG – posterior cingulate gyrus

PCu – precuneus

PIns – posterior insula

PO – parietal operculum

PoG – postcentral gyrus

PP – planum polare

PrG – precentral gyrus

PT – planum temporale

Pu – putamen

R – right

SFG – superior frontal gyrus

SMC – supplementary motor cortex

SMG – supramarginal gyrus

SOG – superior occipital gyrus

SPL – superior parietal lobule

ThP – thalamus proper

TrIFG – triangular part of the inferior frontal gyrus

TTG – transverse temporal gyrus
